# Assessment of Quality of Life of Free Anterolateral Thigh Flap for Reconstruction of Tissue Defects of Total or Near-Total Glossectomy

**DOI:** 10.1155/2020/2920418

**Published:** 2020-10-10

**Authors:** Sanke Zhang, Shuang Wu, Lei Liu, Dandan Zhu, Qiuyu Zhu, Wenlu Li

**Affiliations:** Department of Oral and Maxillofacial Surgery, First Affiliated Hospital of Zhengzhou University, Zhengzhou, Henan 450052, China

## Abstract

**Background:**

The aim of this study was to evaluate quality of life of free anterolateral thigh flap (ALTFF) for reconstruction of tissue defects of total or near-total glossectomy.

**Methods:**

Quality of life was assessed by means of the University of Washington Quality of Life (UW-QOL) and the 14-item Oral Health Impact Profile (OHIP-14), after 12 months postoperatively.

**Results:**

65 of 79 questionnaires were returned (82.27%). In the UW-QOL, the best-scoring domain was “shoulder,” whereas the lowest scores were for “chewing” and “pain.” In the OHIP-14, the lowest-scoring domain was “handicap,” followed by “Social disability” and “Psychological disability.”

**Conclusion:**

Free anterolateral thigh perforator flaps for reconstruction of total or near-total glossectomy defects after cancer resection would have significantly influenced the patients' oral functions and quality of life.

## 1. Introduction

Tongue cancer is one of the common malignant tumors in the oral and maxillofacial region. Although the current treatment presents multiple modes, the prognosis of tongue cancer is not good, and the dominant and hidden lymph node metastasis is also more obvious than other oral malignant tumors. The 5-year survival rate of comprehensive treatment of tongue squamous cell carcinoma in foreign countries is 50%–70% [1–3].

The reconstruction of defects after resection of oral cancer is a challenge because of the critical role of this area both aesthetically and functionally. Radical resection affects all oral functions and can result in subsequent problems. Depending on the location and size of the tongue tumor, radical surgical treatment often affects all oral functions, such as speech, swallowing, chewing, oral rehabilitation, nutrition, and appearance [1]. Now, transfer of free flaps with microvascular anastomoses is the favored method of reconstruction after major operations for oral cancer. The free anterolateral thigh perforator flaps first reported by Song [4] in 1984 has gained popularity in oral cavity reconstructions. It has some advantages, including a long pedicle with a suitable vessel diameter, the availability of different tissues with large amounts of skin, and its adaptability as a sensate or flow-through flap if necessary [5, 6].

Quality of life (QOL) is multidimensional and reflects the patient's point of view, whereas quality of life research reflects the effect of the disease and its treatment on general wellbeing. QOL has become an increasingly important outcome measure for patient's undergoing treatment for a wide array of illnesses. QOL is a global construct that reflects a patient's general sense of wellbeing. It is by definition multidimensional and reflective of the patient's point of view [7]. The aim of this study was to evaluate quality of life of free anterolateral thigh flap for reconstruction of tissue defects of total or near-total glossectomy. These findings could potentially be useful for physicians and patients, while treatment is being planned.

## 2. Materials and Methods

Because this study was retrospective, it was granted an exemption in writing by the Ethical Review Board in the First Affiliated Hospital of Zhengzhou University. From April 2010 through April 2018, a total of 79 patients at the Department of Oral and Maxillofacial Surgery, the First Affiliated Hospital of Zhengzhou University, underwent free anterolateral thigh perforator flaps reconstruction after radical surgery for total and near-total tongue cancer was studied retrospectively. It was granted an exemption in writing by the Ethical Review Board of the Zhengzhou University and the First Affiliated Hospital of Zhengzhou University.

There were 56 males and 23 females, aged 25–70 years old, with a median age of 49 years. The case data were collated, recorded, and retrospectively analyzed to record the primary disease, tongue defect, tissue flap type, and postoperative complications. Case inclusion criteria were advanced tongue or mouth cancer, complete or near-total tongue defect after tumor resection, and free tissue flap to repair tongue defect at the same time. The total tongue defect refers to the defect of the tongue and tongue root tissue, and the proximal tongue defect includes 2/3 in front of the tongue or most of the tongue (2/3 or more). Other inclusion criteria were free flap survived completely; age less than 75 years; no previous or synchronous malignancies; no cognitive impairment; at least 12 months after reconstruction; and patients with recurrence of the disease.

The remaining patients received a formal letter explaining the study, an informed consent form, and the University of Washington quality of life (UW-QoL)/the 14-item Oral Health Impact Profile (OHIP-14). Those patients who did not reply within one month received a reminder. Patients who were not able to fill in the questionnaires themselves, e.g., due to dementia or language were excluded from the study. Patient characteristics are summarized in Table 1.

### 2.1. Questionnaires and Data Collection

Although many generic QOL instruments have been developed over the past 30 years, the University of Washington quality of life (UW-QoL) [8] and OHIP-14 consists of 14 items divided into 7 domains: functional limitation, physical pain, psychological discomfort, physical disability, psychological disability, social disability, and handicap. Each item is scored as 0 = never; 1 = hardly ever; 2 = sometimes; 3 = fairly often; and 4 = very often. The domains are scored on a scale ranging from 0 (best) to 100 (worst). The higher the score, the poorer the patient's state of health. The standard OHIP-14 is available in Chinese and has been validated for a Chinese population [8]. The UW-QoL scale is filled in by the patient and provides a broad measure of QoL for patients with head and neck cancer with good acceptability, practicality, validity, reliability, and responsiveness. The questionnaire is composed of 15 domains: 12 are disease-specific (pain, appearance, activity, recreation, swallowing, chewing, speech, shoulder, taste, saliva, mood, and anxiety), and 3 are global questions. Each of the 12 questions included has 3–6 choices of response. The domains are scored on a scale ranging from 0 (worst) to 100 (best). The standard UW-QoL is available in Chinese and has been validated for a Chinese population [8] according to the methods of our past research [9].

## 3. Results

### 3.1. Patient Characteristics

The patients' demographic and TNM/clinical stages are listed in Table 1. Of the 65 patients who completed questionnaires, patients were staged according to the 2010 American Joint Committee on Cancer (AJCC) staging system; their main clinical characteristics are listed in Table 1. In our research, more than half our patients had had little education, 18 patients could not read or write and needed help to complete the questionnaire, 5 patients were orphans, and 7 patients lived alone.

But even in this, 65 of the 79 questionnaires (82.27%) were completed, SF-36 and OHIP-14, at one or two time points during the treatment and follow-up periods. The time needed for completing both questionnaires is very acceptable and makes it feasible to use them in clinical studies. Data for the SF-36 and OHIP-14 scales at 12 months after TFFF are shown in Tables 2 and 3. In TNM stages research studies, we found that most patients had T3-T4 stage tumors and only a few T1 and T2 stages. This may be because patients with T1-T2 stage tumors had small tumors that did not need AFFF reconstruction.

### 3.2. UW-QOL

The scores for the 12 disease-specific domains and the importance of each domain are shown in Table 2. At the top of the list of high-scoring domains were shoulder and anxiety. 15 patients scored 80% or more for anxiety (mean (SD) 70.5 (6.7) points). The best-scoring domain was shoulder, with a mean (SD) score of 78.6 (11.3) points. The worst score was for chewing, with a mean (SD) score of 41.2 (13.2) points. All patients were dissatisfied with chewing, speech, recreation, and pain. In the selection of the most important of the 3 domains, chewing was considered most important, followed by speech and swallowing. The domains pain, swallowing, and taste were the least important to patients.

### 3.3. OHIP-14

Distributions of OHIP-14 domain scores at presentation are shown in Table 3. The best mean (SD) domain scores for the complete group were 30.2 (5.6) for handicap, 43.8 (8.7) for psychological disability, and 45.2 (11.2) for social disability. The highest scores were for psychological disability and physical pain.

## 4. Discussion

Oral cancer is one of the most common malignant tumors in the body. The incidence of tongue cancer in oral cancer is the highest. Recent studies at home and abroad have shown that the incidence of oral cancer is on the rise in the world [10], and the age of the disease tends to be younger [11]. Tongue cancer has the characteristics of high malignancy, high local recurrence rate, high cervical lymph node metastasis rate, and often endangers patients' lives. Therefore, the best choice is surgical radicalization. At present, most of the surgery is based on comprehensive treatment [12]. At present, it is generally acknowledged that free flaps transfer with microvascular anastomosis is the favored method for reconstruction after major oral cancer surgery [13]. Yang et al. first made the radial free forearm flap (RFFF) in 1981, which was a major breakthrough in reconstructive surgery and appreciated in this type of reconstruction. It is a free tissue transfer characterized by thinness, pliability, a long vascular pedicle, and contour ability and maintains consistent volume and surface area over it. AFFF helped to shift focus away from simple coverage of defects towards minimizing morbidity at the donor site, refining the flap, selecting the most appropriate donor tissue, reducing bulk, and reconstructing defects in a functional, three-dimensional manner, and it was recognized as the method of choice for reconstruction of soft tissue defects in the oral cavity and oropharynx, especially in tongue cancer.

For patients with total and near-total glossectomy, oral feeding and speech function are largely dependent on flap reconstruction to restore tongue morphology and dynamic function. Kimata et al. [14] divided the shape of the reconstructed tongue into the oral cavity into four types: bulge type, semi-uplift type, flat type, and concave type. Among them, the bulging type and the semi-uplift type are basically in contact with the upper jaw when the mouth is closed, and the gap between the oropharynx is narrow, which is conducive to the recovery of eating and language function. In order to the reconstructed tongue to reach a bulge and a semiembossed configuration, the repaired tissue is required to have a sufficient and relatively constant volume. Therefore, the key point of functional recovery after total tongue and near-total tongue reconstruction is the amount of flap tissue. The free anterolateral thigh flap can provide a large amount of soft tissue and can carry part of muscle tissue, which is beneficial to recovery of oral function in postoperative patients.

In the assessment of any therapy, particularly for cancer patients, analysis of QOL represents the ultimate step of the evaluation process. QOL has recently become a constant preoccupation in the assessment of any therapy in oncology. Some authors describe QOL as welfare state along with patient's satisfaction. Other authors relate QOL as the difference between the patient's expectations and what they can really perceive. Assessment of QOL can now be used in an attempt to improve treatment outcomes, and to measure success or failure in cancer treatment has been survival, understood as a period free of disease. Completion of questionnaires can help put into context what other patients report as their outcome after intervention allowing greater patient-doctor interactions and understanding. It also allows expressions of concern that the patient is otherwise reluctant to mention. Nevertheless, few studies have assessed QOL in our field of interest. In our study, we used the UW-QOL and OHIP-14 questionnaires to assess the postoperative QOL of these patients and the possible relation to surgery.

The UW-QOL measure was chosen as the oral-specific questionnaire that provided a broad measure of QOL for patients with tongue cancer with good acceptability, practicality, validity, reliability, and responsiveness. Language is simple, but because individual response options are provided for each question rather than using a standardized scale, the reading burden is high. Each item is scored from 0 to 100, with higher scores indicating better QOL, resulting in a summary score of 0–900 for the disease-specific items, and each question has 3–6 response options. It is brief and appropriate, so it can be used on a regular basis with low cost. It presents an additional module, which evaluates emotional status and anxiety. In our study, many authors have chosen to use the UW-QOL questionnaire [8], and the highest score in the UW-QOL subscales was in the domain shoulder. The mean (SD) score was 78.6 (11.3), which indicated slight damage in the shoulder domain. As well as shoulder and patients scored high for anxiety (70.5 (6.3)) and taste (65.4 (7.5)), which indicates that the operation, had little effect on the functions of taste and anxiety. As well as the domain of pain, chewing, and swallowing were lower than others because of tongue cancer characteristics, patients reluctant to communicate with other, especially when sharp stimulus contained sharp teeth or dentures around primary tumor in our study.

We used the Chinese version of the OHIP-14, which has been translated and validated for use in Hong Kong and China [15]. The best mean (SD) domain scores for the complete group were 30.2 (5.6) for handicap, 43.8 (8.7) for psychological disability, and 45.2 (11.2) for social disability. The highest score was for physical disability (71.2 (9.2)), and no patients scored 40%. For the physical disability questions, we asked “Has your diet been satisfactory because of your teeth, mouth, or dentures?” and “Have you had to interrupt meals because of your teeth, mouth, or dentures?” No patient was satisfied with the degree of physical disability. Loss of teeth greatly weakened the patients' oral function.

Total tongue resection and the extent of local lesion resection are large; patients with malignant tumors need to undergo unilateral or bilateral neck dissection, and free flap transplantation at the same time has the problems of complicated operation, large trauma, and long duration. In addition, such patients usually require tracheotomy, and the possibility of postoperative pulmonary infection is high. At the same time, due to poor tongue swallowing function, the possibility of aspiration after surgery is also high, resulting in inhalation pneumonia, leading to a series of postoperative complications. Moreover, patients with postoperative head brakes need to rest in bed for a long time, which is easy to cause complications for patients with poor cardiopulmonary function. Therefore, the general anesthesia should be used to evaluate the general condition of the patient before surgery, and the preoperative examination should be improved to deal with adverse reactions.

Because of the rich supply of lymph and blood and tongue cancer more prone to lymph node metastasis and blood metastasis, cervical dissection must be carried out during operation. Worse QOL has been found in patients with cervical dissection operation compared to those who did not have it [16, 17], related to the fact that they are patients in a more advanced stage. The cervical dissection along with the scar consequence of the surgical reconstruction with a myocutaneous pediculed flap is a combination that in a most important way influences in patient's complaints on esthetic and pain in the reconstructed location [18]. Little shoulder dysfunction has been reported with unilateral selective neck dissections (level I–III/IV) as compared with no dissection. During our study, significantly worse shoulder function was also found if selective neck dissections were bilateral or extended to level V.

There were limitations that could influence the result of our findings. We described oral cancer in the study population at one point in time, and so could not fully assess its impact on patients' QOL over the whole postoperative period. Under these circumstances, QOL assessment is quite a new area, and the emphasis is placed on clinical practice and research. Considerable effort should therefore be put into it. QOL should be acknowledged as an important outcome, together with traditional biomedical outcomes.

## 5. Conclusion

In total and near-total tongue cancer postoperative free flap for reconstruction, QQL assessment is quite a new area, and the emphasis is placed on clinical practice and research. Therefore, considerable effort should be paid to this area. QQL should be acknowledged as an important outcome parameter, along with the traditional biomedical outcomes. Clinically, QQL should be used as a part of tongue cancer treatment, and this should be considered for surgical planning. ALTFF for reconstruction of defects of tongue significantly influenced QOL.

## Figures and Tables

**Table 1:** Patients' demographic and TNM/clinical stages (*n* = 65).

Patients' demographic and stages	Number of cases	(%)
Male	56	86.2
Age < 65 *Y*	47	72.3
Karnofsky score <70	43	66.2
Primary tumor		
T1	4	6.2
T2	8	12.3
T3	31	47.8
T4	22	33.8
Regional lymph nodes		
N0	27	41.5
NI	12	18.5
N2a	8	12.3
N2b	9	13.8
N2c	6	9.2
N3	3	4.6
Distant metastasis		
M0	61	93.8
M1	4	6.2
Clinical stages		
I	3	4.6
II	10	15.4
III	29	44.6
IV (IV*a*IV*b*IV*c*)	23	35.4
Pathologic diagnosis		
Squamous cell carcinoma	56	86.2
Other carcinoma	9	13.8

**Table 2:** Means of scores of items and scales of University of Washington Quality of Life questionnaire.

Domain	Number of cases (*n* = 65)		
	Mean (SD)	Median (range)	Percentage with score 80+
Pain	42.7 (4.5)	43 (20–65)	0
Appearance	56.8 (5.4)	58 (40–82)	4
Activity	65.3 (10.7)	66 (35–86)	9
Recreation	56.1 (6.2)	57 (30–70)	0
Swallowing	43.8 (9.3)	44 (15–66)	0
Chewing	41.2 (13.2)	42 (10–65)	0
Speech	53.2 (6.4)	54 (0–70)	0
Shoulder	78.6 (11.3)	79 (60–88)	15
Taste	65.4 (7.5)	66 (10–75)	0
Saliva	61.2 (8.5)	63 (30–85)	8
Mood	61.9 (7.9)	62 (40–75)	0
Anxiety	70.5 (6.7)	71 (45–86)	12

**Table 3:** Means of scores of items and scales of Oral Health Impact Profile-14 questionnaire.

Domain	Number of cases (*n* = 65)		
	Mean (SD)	Median (range)	Score 40 or fewer (%)
Functional limitation	52.3 (4.5)	53 (20–66)	11
Physical pain	55.3 (5.4)	56 (15–82)	14
Psychological discomfort	51.7 (10.1)	53 (20–73)	18
Physical disability	71.2 (9.2)	72 (30–80)	8
Psychological disability	43.8 (8.7)	44 (15–66)	36
Social disability	45.2 (11.2)	46 (10–60)	22
Handicap	30.2 (5.6)	30 (0–45)	55

## Data Availability

The data used to support the findings of this study are included within the article.
